# Memory distrust and imagination inflation: A registered report

**DOI:** 10.1371/journal.pone.0297774

**Published:** 2024-02-07

**Authors:** Iwona Dudek, Romuald Polczyk

**Affiliations:** 1 Doctoral School in the Social Sciences, Jagiellonian University, Kraków, Poland; 2 Institute of Psychology, Faculty of Philosophy, Jagiellonian University, Kraków, Poland; University of Sussex, UNITED KINGDOM

## Abstract

Imagination inflation happens when a person’s subjective confidence that an event has occurred increases after they imagine it occurring. In this project, our primary aim is to test whether memory distrust is related to the imagination inflation effect in people who are aware of the discrepancies between their own memories and what they have imagined. Our secondary purpose is to investigate whether the influence of memory distrust on imagination inflation is moderated by traits that are described as *disengagement from reality* and to test whether memory distrust mediates the relationship between self-esteem and imagination inflation. In a three-step procedure, participants (*N* = 300) will assess their confidence that a list of childhood events happened to them; then, they will imagine three of these events and reassess their confidence. Half of the participants will undergo a memory distrust induction procedure. In order to sensitize participants to discrepancies, some of them will be given cues about the source and/or perspective of the imagined events.

## Introduction

Having spent months in therapy, Sheri J. Storm remembered being sexually abused by her father as a child and being forced to engage in bestiality and satanic rituals. It turned out that she was not the only person who remembered similar childhood events under the care of the same therapist. After some time, she realized that the abuse had never happened [1]. Sheri J. Storm’s therapy focused on the revelation of alleged traumatic childhood events that had been repressed from consciousness because of their harrowing nature. The theory of repressed memories has little scientific basis because no studies have provided strong support for its validity [2, 3]. Unfortunately, in the course of therapy, many therapists engage clients in exercises such as repeatedly imagining scenarios (often suggested by the therapist) that might have happened [4, 5].

Today, it is known that it is possible to create false memories of entire events in people just by asking them to imagine these events. Imagination enhances confidence that the imagined event occurred in real life. This effect is called imagination inflation [6] and can be classified as a subtype of confabulation that occurs when individuals assert and genuinely believe that an event occurred, despite it never actually taking place [7]. A typical procedure for studying this that was developed by Garry et al. [6] is as follows: participants are presented with the Life Events Inventory (LEI), which consists of 40 childhood events; on an 8-point scale, they are then asked to rate the likelihood of these events. Two weeks later, participants imagine four of the eight target events that are rated as low-likelihood events. They are then presented with a cover story that their LEI sheets have been misplaced, and they are asked to complete the LEI a second time. For the target events, the fact that participants had imagined them inflated their confidence that these events had occurred in their childhood. Several subsequent experiments replicated these results, e.g., [8–11].

Not only during recovered memory therapy, but also during interrogation, people can be exposed to the influence of suggestion, which can result in confabulation. This is illustrated by the case of Paul Ingram, who confessed to sexually abusing his daughters and believed that he was the leader of a satanic sect. Paul Ingram was forced to admit that his daughters would not lie about sexual abuse and he was convinced that his memories of the horrific acts he had allegedly committed could have been repressed. His trust in his own memory was undermined. Ingram began to confess, but without giving details of the crimes. As a result of visualizing the scenes suggested by the detectives, he gave details of crimes that never really happened (detailed description of this case: [12, 13]).

Memory distrust is known to increase susceptibility to suggestion. This term refers to a condition in which individuals experience a deep sense of doubt or skepticism towards their own memory. As a consequence, they become highly vulnerable to depending on external cues and suggestions as a means of shaping their beliefs or memories [14]. Two types of memory distrust are distinguished: trait and state [15]. Trait memory distrust is stable over time and refers to habitual distrust of one’s own memory ([16, 17]. It has two aspects. The first, which used to be the main focus in research on the role of memory distrust in the development of various forms of memory distortions (e.g., [15, 18, 19]), pertains to the subjective belief of an individual that they have a tendency to make omission errors, namely the apprehension of forgetting something [17]. The second aspect, which is moderately correlated with the first, relates to the subjective belief of an individual that they have a tendency to make commission errors, such as confusing imagination or dreams with reality [17, 20].

In turn, state memory distrust is related to a specific situation in which one experiences a lack of trust in one’s own memory. In a forensic context, this situation is often related to interrogation: for example, a police officer can challenge one’s memory by subtle and manipulative questions [21, 22]. Memory distrust can be also invoked because of interviewee health issues, such as alcoholism, dementia, or dissociative-related memory problems [23].

Moreover, in certain clinical cases like obsessive-compulsive disorder, individuals experience persistent doubt regarding their memories of past actions, leading them to engage in repetitive checking behaviors [24]. There are even instances of what are known as non-believed memories, where people remember an event (have a vivid recollections), but they consciously have low or no belief in it [25].

Gudjonsson claims that, under specific conditions, memory distrust can result in false confessions, especially of the pressured-internalized type, which might be accompanied by false memories of how the crime in question was committed (confabulation) [26]. He provided a detailed description of several cases in which memory distrust played a significant role in internalized false confessions, and he developed a heuristic model whereby contextual risk factors (e.g., isolation), personal vulnerability (including trait memory distrust) and acute mental states (including state memory distrust) lead to the development of memory distrust syndrome [21, 22, 26]. In an experimental study in which five different interrogation techniques were used, including suggesting memory problems, memory distrust was found to significantly correlate with false confession tendency [15].

Memory distrust has also been studied in the context of other subtypes of confabulation. Van Bergen et al. [27] found that individuals suffering from memory distrust are prone to accepting misinformation; however, these individuals had similar levels of interrogative suggestibility and compliance traits as those who did not suffer from memory distrust. Subjective memory ratings have been negatively correlated with compliance, but no significant correlation has been found between subjective memory ratings and false recollections (i.e., critical lures) elicited by the DRM task [28] and interrogative suggestibility [19]. Greater memory distrust is associated with a higher frequency of false memories, non-believed memories (NBM, i.e., vivid recollection of an event whose occurrence is no longer believed as much) and lower self-esteem [29].

Van Bergen and Jelicic [27] conducted an experimental study on the relationship between memory distrust and imagination inflation. First-year university students were exposed to a manipulation of memory distrust by providing feedback in a memory task that involved recalling eight events from their childhoods. Feedback was positive, negative or not provided at all and was not related to actual performance on the task. The positive feedback group was falsely informed that they had performed very well compared to other students, who usually could not even remember very much. The negative feedback group was asked why they had only been able to write down a few memories. If a participant recalled all eight events, they were asked why their memories had so few details. In addition, participants in this group were asked whether they might have had a concussion or other neurological condition that could explain their poor performance. Then, the participants were subjected to an imagination inflation procedure. This study found that manipulating memory distrust did not enhance the effect of imagination inflation.

### Overview of the present study

Although Van Bergen and Jelicic’s [30] study suggests that there is no relationship between memory distrust and susceptibility to the imagination inflation effect, in this project we still want to explore this topic. However, we assume that the relationship between memory distrust and imagination inflation will be revealed in people who are aware the discrepancies between actual childhood memories and imagined events. In addition, our second goal is to examine the interactional effect of disengagement from reality traits [31] on the relationship between memory distrust and the imagination inflation effect. Finally, since how a person perceives memory capacity is related to how they perceive themselves, we would like to test whether self-esteem affects the magnitude of the imagination inflation effect through memory distrust. In subsequent sections, we provide detailed justification for all hypotheses.

#### Sensitization to discrepancies

In research on another type of susceptibility to suggestion, namely the misinformation effect, participants receive misleading post-event information. According to discrepancy detection theory [32], people who are able to distinguish discrepancies between the original event and the post-event information are less susceptible (though not completely immune [33, 34]) to the misinformation effect. The imagination inflation paradigm shares similarity with the misinformation paradigm to some extent: participants themselves generate misleading post-event information when they imagine childhood events. The information acquired while imagining these events increases confidence that they took place, thus causing an inflation effect. Similar to research on the misinformation effect, it has been shown that by giving people cues to help them distinguish imagined events from actual childhood events, they are less likely to experience imagination inflation [35].

By examining the misinformation effect, it is possible to check whether a participant, while answering the final memory test questions, has in memory both the original information and the information suggested in the material with which they became acquainted later [36]. It has been found that some people are aware of these discrepancies but still succumb to the misinformation [33, 34]. Diagnosis of discrepancy awareness involves asking participants who have completed the memory test to state whether a particular detail, e.g., the color of a thief’s car, was mentioned in the original and/or follow-up material and to write which detail was in each piece of material (e.g., yellow car in the original material and red car in the follow-up material). A similar procedure can be used when testing another type of susceptibility to suggestion, namely interrogative suggestibility [37]. Identifying participants who are aware of the discrepancy seems to be key to studying the relationship between memory distrust and proneness to memory distortion, since only individuals who have both the original and the suggested information in memory can decide whether to rely on their own memory or the suggested information when making a decision in the post-test.

It should be noted that that there is a longstanding debate about the mechanisms of the misinformation effect; in particular, about what happens to the original memory trace. Many theories have been proposed: the parallel traces theory [38, 39]; the CHARM model [40]; the fuzzy trace theory [41]; the activation-based framework [42]; retrieval-induced forgetting [43]; explanations based on the source monitoring idea (e.g., [44–51]), etc. The fact that there are “diverse routes leading to the misinformation effect” seems to be widely accepted now [52]. In the present work, we do not deny that various distortions of the memory process may moderate the impact of misinformation on memory reports. We merely want to explore some ideas which are currently less studied, namely the possibility that some participants do in fact remember (and have perfect access to) the original information as well as the misinformation contained in the post-event material, yet for some reason they give answers consistent with the latter.

In the context of imagination inflation with childhood events, it would be difficult to design a procedure to check whether participants are aware of discrepancies between actual childhood memories and imagined ones when completing the LEI post-test. Therefore, it was decided to introduce an experimental manipulation to sensitize participants to discrepancies. This manipulation was originally designed by Sharman et al. [35] to immunize against the imagination inflation effect. In Sharman et al.’s study, participants were given cues to help them resist the effect of imagination inflation. First, the subjects filled out the Life Events Inventory (LEI), thereby assessing their confidence that a list of childhood events had happened to them; then, they imagined some of these events; finally, they filled out the LEI a second time, again evaluating their confidence in the events. Participants were assigned to one of four groups. The first group received a cue about the source of their memories (they were instructed to imagine events from a first-person perspective), while the second group received a familiarity cue (a plausibility questionnaire, which contained the same events as the LEI but in a different context, before completing the LEI 2). The third group received both cues, and the fourth group received no cues. Single cues were found to be insufficient to protect against the imagination inflation effect, and only those given two cues were immune to it. This means that additional cues can sometimes protect people from becoming more confident that imagined fictitious events were genuine experiences.

This manipulation of cues used by Sharman et al. [35] is based on two postulated mechanisms of imagination inflation, one of which is source misattribution [53]. Imagination inflation appears to be caused by internal misattributions (a memory is treated as something real rather than as something imagined). As a consequence, after an imagination session, people judge the imagined event as more authentic and rate its likelihood as higher. The same mechanism is responsible for the misinformation effect, which results from erroneous external source attributions (the memory is attributed to the original event instead of to the post-event information) [32].

A cue regarding the source of a given memory seems to reduce the imagination inflation effect [35]. Such a cue may be the perspective from which childhood events are imagined. It is well established that newer memories are more often recalled from a first-person perspective than from a third-person perspective, while older memories are more often recalled from a third-person perspective than from a first-person perspective (for a review, see [54]. Thus, one can derive the assumption that the phenomenological characteristics of childhood events imagined from a third-person perspective are more likely to correspond to the phenomenological characteristics of reconstructed real childhood memories compared to those imagined from a first-person perspective [55]. As in previous research [10, 35], we assume that this change in perspectives should act as a source cue that alerts participants to the differences between their imagined memories and their actual childhood memories.

The second explanation of imagination inflation assumes that familiarity can be used to evaluate the source of a memory [53] and that imagination inflation occurs as a result of misattribution of familiarity [56]. This means that more-frequently experienced events are more fluidly processed and spring more easily to mind, which can result in an increased likelihood of claiming that an event was actually experienced [57, 58]. As a result of this smoother processing, there can also be an increase in confidence that an event actually occurred, i.e., there is an inflation of the imagination effect [35]. In our study, the way to sensitize participants to familiarity will be the same as in Sharman’s study: it will involve providing subjects with a source to which they can attribute their sense of familiarity of LEI events. Before completing the LEI post-test, some participants will complete a questionnaire containing the exact same events found in the LEI, but in a different context (i.e., a question about how plausible it is that a typical Pole who is about the same age as the participants had certain childhood experiences by age 10). These individuals will make a judgment based on the ease with which the events come to their mind. Giving participants a source to which they can attribute the increased cognitive accessibility of events should sensitize them to the discrepancy between their real childhood memories and imagined ones.

#### Individual differences: Disengagement from reality

Individual differences in susceptibility to imagination inflation seem to be predicted by *disengagement from reality* traits [31], although the research findings are inconclusive. Thus, dissociation–conceived as differences between the ability to discriminate and integrate memories, fantasies, motivations, and actions in awareness [59, 60]–has been shown to correlate positively with imagination inflation [31, 61, 62]; however, other studies have not confirmed this [9, 63]. The ability to visualize or create mental imagery positively correlates with imagination inflation [9]; however, as with dissociation, other studies have not confirmed this relationship [31, 61, 63]. An association between imagination inflation and hypnotic susceptibility has been found [61].

The aforementioned research was conducted in the paradigm of guided imagination inflation. However, research on false autobiographical memories and their correlates related to traits of disengagement from reality is not limited to this experimental manipulation. For example, Hyman and Billings [64] investigated the possibility of students developing false childhood memories and the role of individual differences related to the formation of these memories. Students were asked about several true childhood events (provided by a family member) and one false event (made up by the experimenter) that a family member confirmed the subject had never experienced as a child. Students described all events in two interviews. When they could not recall an event (real or false), they were encouraged to think about it and to try to imagine it. About 25% of the students created false childhood memories, and proneness to both dissociation and fantasy were found to be positively related to false memory creation. Using a similar methodology, Porter et al. [65] showed a link between the formation of false autobiographical memories and proneness to dissociation; however, in an experiment using guided visualization that was conducted by Paddock et al. [66], dissociation was not related to false memory. Horselenberg et al. [67] found a negative correlation between, on one hand, the ability to discriminate between real autobiographical events that participants themselves had documented six months earlier and, on the other hand, false events and fantasy proneness, but not dissociation, absorption, or suggestibility.

#### Memory distrust: In search of mediators of the relationship between self-esteem and susceptibility to imagination inflation

We will test whether self-esteem affects the magnitude of the imagination inflation effect through memory distrust. Self-esteem may exacerbate memory distrust because an individual’s perceived memory capacity is related to how they perceive themselves, i.e., their self-esteem. It is difficult to clearly define the direction of this relationship. It has been shown, for example, that one predictor of older people’s perceptions of their subjective memory ratings (that is, ratings of their own memory abilities in different types of situations) is their self-esteem [68]. These authors stress, however, that perhaps this self-esteem depends on how people perceive their own memory, and the correlational nature of the research does not allow causal conclusions to be drawn. Self-esteem is negatively related to the Cognitive Failure Questionnaire (CFQ) [69], a self-report measure of memory functioning.

The sociometric theory of self-esteem assumes that self-esteem acts as a measure of an individual’s relational value in social groups [70, 71], and this may explain why low self-esteem may increase memory distrust. According to this view, low self-esteem is linked to social anxiety, social alienation, and perceived maltreatment by society, among other factors [72, 73]. It has been indicated that low self-esteem may result from more frequent negative feedback from others, and this may be associated with increased levels of memory distrust [29]. In addition, people with low self-esteem, who are more likely to experience rejection, may take negative feedback more easily. According to Ridley and Gudjonsson [74], they may not be able to cope with the demands of interrogation because it could make them more susceptible to developing memory distrust. Previous research has found a direct link between low self-esteem and suggestibility. Saunders [75] found that participants with low self-esteem were more likely to succumb to misinformation. On the other hand, self-affirmation techniques that rely on writing down life achievements combined with positive feedback on performance in a memory task can immunize against misinformation [76, 77]. Similarly, low self-esteem has been found to increase interrogative suggestibility [78–81] (but see also: [82, 83], for a review; [84]). Recently, in correlational studies, Zhang et al. [29] asked participants to indicate how often they experience false memory and non-believed memories (NBM). They found higher memory distrust was associated with a higher frequency of false memories, NBM and lower self-esteem. In a mediation analysis, they found a significant indirect effect of self-esteem on false memory (Study 1a and Study 2) and NBM (Study 2) through trait memory distrust.

#### Hypotheses

The aim of our study is to test hypotheses in three areas. First, we will examine the relationship between trait memory distrust (MD) and imagination inflation (II). Second, we will explore individual differences that might moderate the effect of MD on susceptibility to II. Finally, we will investigate the mediating role of MD in the relationship between self-esteem and II.

***Hypothesis 1*** concerns the relationship between the trait of memory distrust and the effect of imagination inflation. Our predictions are different for each of the two aspects of memory distrust. In terms of the subjectively experienced susceptibility to make memory omission errors, we predict that there will be no correlation between them and II because previous research has shown that this aspect seems to correlate weakly or not at all with the formation of memory distortions (cf. no significant correlation between trait MD and interrogative suggestibility and false recall [18, 19]) (***Hypothesis 1a***). In contrast, we assume that subjectively experienced susceptibility to make commission errors will be positively correlated with II (***Hypothesis 1b***) because errors of commission are more closely linked to the development of false beliefs and false memories than errors of omission [21]. A recent study shows that when compared to the individually perceived inclination to commit memory omission errors, individually perceived inclination to commit commission errors proved to be a better predictor of individuals’ ratings of autobiographical belief (i.e., individuals’ belief in the occurrence of specific autobiographical events). Specifically, individuals who were concerned about having distorted or false memories were more inclined to report events with lower belief ratings than those who were less concerned [17]. It is therefore likely that people who believe that they are susceptible to memory commission errors will rely more on imagined events, resulting in a greater II effect.

We believe that the relationship between memory distrust and imagination inflation will be revealed by an experimental manipulation involving the induction of a transient state of MD and sensitization to discrepancies (cf. Hypothesis 2). In ***Hypothesis 2***, we assume that the moderating effect of MD should be revealed in groups that are “sensitized to discrepancies” by at least one method (i.e., either source cue, SC [first-person perspective in an imagination exercise], or familiarity cue [FC; questionnaire asking, “How plausible is it that the typical Pole who is about your age had certain childhood experiences by age 10?”], or both cues). In these groups, we expect that participants with MD induction will experience a greater II effect compared to those without MD induction (as revealed by the respective interaction being statistically significant).

Specifically, ***Hypothesis 2a***, which concerns SC and FC, states that the effect of MD induction on II will be present in conditions where at least one discrepancy sensitization manipulation (SC and/or FC) is used, but it will not be present in the condition with no discrepancy sensitization manipulation.

The ***Hypothesis 2b***, which concerns the additive effect of SC and FC, states that II will be greater in the group that will be subjected to MD induction and will receive FC and SC than in the group that will be subjected to MD induction and will not receive any of these cues. We assume that providing two cues will increase the chances of participants noticing discrepancies and will sensitize them more than providing a single cue.

In ***Hypothesis 3*** we predict that the influence of MD on II will be moderated by the tendency to disengage from reality, which is operationalized as high visual imagery vividness, tendency to use a visual cognitive style, strong imaginative-fantasy ability, high absorption, and tendency towards dissociation. We predict that this tendency to disengage from reality may not be beneficial for memory functioning because, in the II paradigm, time is scheduled for participants to imagine the event. Participants with these traits may find it difficult to distinguish between imagined ideas and real memory traces, and the imagined information will be more vivid and accessible than real memories.

One of the traits addressed by this hypothesis is absorption, i.e., the disposition to become deeply involved in experiences, both real and imagined, while effectively excluding other sources of information [85, 86]. It seems reasonable to assume that people with high levels of this disposition may have difficulty distinguishing between real and imagined events. Because absorption is sometimes considered a fundamental aspect of dissociation, a similar assumption is made for the latter as it is often seen as a core element in the typical dissociative process [87]. Dissociation in its non-pathological form is sometimes experienced in everyday life and manifests as deep focus and narrowed attention, resulting in less awareness of internal states or external activities [88].

The three traits relating to imagination (imagery vividness, visual cognitive style, and imagination immersion) do not appear to be the same [89]. They are defined differently: imagery vividness is usually understood as some sort of ability to create vivid mental images. For example, Tulving, McNulty, and Ozier [90] stated that imagery vividness is “the ease with which you can picture something in your mind” (p. 242). Similarly, Quilter, Band, and Miller [91] stated that “Vividness is the extent to which the client can mentally create images and experiences that are clear, sharp, and detailed” (p. 162). Imaginative involvement consists in experiencing some imagined events with an “almost total immersion in the activity, [and] with indifference to distracting stimuli in the environment” (p. 5) [92]. Cognitive style refers to the preferred style of information processing [93]. Because of these differences in meaning, we decided to include all of them as measures of some kind of disengagement from reality. All the hypotheses that relate to each of them separately will be tested.

**Fig 1:**
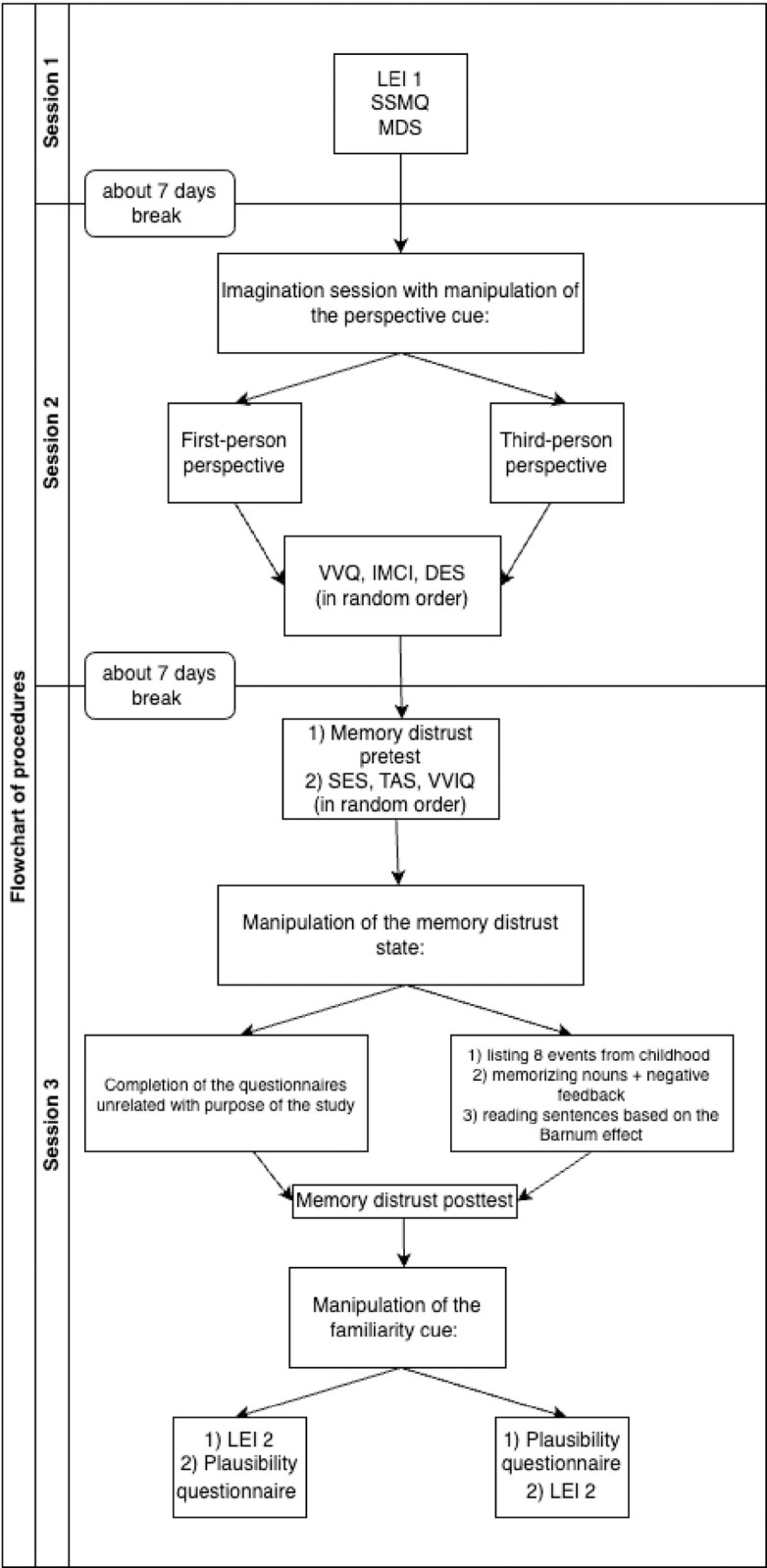
Conceptual mediation model.

Finally, in ***Hypothesis 4*** we predict that the. efficacy of memory distrust induction will be moderated by trait self-esteem in such a way that this efficacy will be higher among participants with low self-esteem. We expect this because, in the case of people with low self-esteem, any external negative feedback should have a particularly devastating effect on their self-trust, including memory distrust. After all, negative feedback just confirms what such persons believe about themselves. In contrast, people with high self-esteem may be more resistant to negative feedback (at least in the case of stable high self-esteem, perhaps less so when it is fragile). In sum, this would mean that self-esteem has a negative impact on the final variable, namely confidence that the imagined events occurred in real life, because this depends on memory distrust, which in turn depends on self-esteem as a factor moderating the efficacy of the manipulation that induces memory distrust. The proposed model is illustrated in Fig 1.

## Methods

Approval from the Research Ethics Committee of a higher education institution in Cracow, Poland was obtained (no KE/55_2022). Written consent for participation will be obtained from all persons in the study prior to data collection.

### Power and sample size analysis

To identify the relevant sample sizes, a set of a priori power analyses was performed. Each of the analyses was performed for a power of 80% and alpha level of 0.05. Three analyses were performed for each problem: for the small, medium, and large effect size. Except for the analysis of the indirect effect (mediation analysis), which was based on information and tables provided by Fritz and MacKinnon [94], the analyses were performed by means of G*Power 3.1 software [95]. Screenshots from G*Power are provided as supplementary materials.

The first analysis focused on the within-subject effect of imagining events on confidence rating. Repeated-measures ANOVA was chosen as the method of analysis. A rather moderate-strength correlation of 0.5 between the pre- and post-test was assumed. For the small, medium, and large effect sizes (f = 0.10. 0.25, and 0.40), the required sample sizes were 199, 34, and 15 respectively (it should be noted that the conventional effect sizes are for between-subjects rather than within-subjects design [96]).

The second power analysis looked at the sample sizes required to detect between- and within-subjects interactions; this is needed in the case of the hypothesis that MD induction causes a greater change in the pre- versus post-tests of II. Again assuming small, medium, and large effect sizes (f = 0.10. 0.25, and 0.40), the sample sizes required to detect interactions, as indicated by G*Power were, 200, 34, and 16, respectively.

The third problem concerned the moderation analysis. Technically, detecting moderation in a regression analysis framework consists in analyzing whether the product of the independent variable and the moderator is a significant predictor of the dependent variable after controlling for the independent variable and the moderator. This can be analyzed as a statistically significant increase in R-square by means of G*Power. Effect sizes of f^2^ equaling 0.02, 0.15, and 0.35 were assumed, resulting in sample sizes of 395, 55, and 35 participants, respectively.

Finally, the power analysis for the indirect effect (mediation analysis) was based on existing tables: Fritz and MacKinnon [94] provided information about the sample sizes needed for 80% power for tests of mediation for four effect sizes concerning the predictor > mediator and mediator > dependent paths: 0.14, 0.26, 0.39 and 0.59. When both paths are close to the smallest effect size, the required sample size is 558. An effect size of 0.26 for both paths requires a sample size of 162, while an effect size of 0.39 requires a sample size of 78.

Given the resources available, we decided to use a sample size of 300 participants as this would allow us to detect at least medium-sized effects in all analyses, and large ones in some of them.

### Participants

The experiment will involve 300 participants over the age of 18. They will receive 60 PLN (about 8 EUR) for participation. Participants will be recruited through posters and advertisements on social media and job sites. Previous participation in memory distortion research will preclude candidates from taking part in the study.

### Design

The design will be a mixed design: 2 (memory distrust induction: yes *vs*. no) × 2 (source cue: first-person perspective *vs*. third-person perspective) × 2 (familiarity cue: plausibility questionnaire first *vs*. LEI 2 first) ×2 (event type: imagined vs. not imagined) × 2 (LEI completion time: LEI 1 (pre-test) *vs*. LEI 2 (post-test)). The between-subjects variables will be memory distrust induction, the perspective cue (whether the participant imagines events from a first-person or third-person perspective), and the familiarity cue (whether the participant is the first to complete the LEI 2 or the plausibility questionnaire). The within-subjects variables will be time of completion, LEI, and event type. The counterbalance is that each participant will imagine a randomly selected three of the six target events. The other three events will serve as controls. The order of presenting these events will be counterbalanced across subjects.

### Materials

**Life Events Inventory (LEI)** consists of 20 events, translated and adapted to Polish culture by the first author; it is similar to Marsh et al.’s [55] questionnaire. Six of the events are critical target events; three events are unrealistic and are included to ensure that participants pay attention and provide credible answers. Subjects will rate their confidence that the events happened before the age of 10 on a Likert scale from 1 (definitely did not happen to me prior to age 10) to 8 (definitely did happen to me prior to age 10). The final result will be computed as the mean ratings of the three target events.

**The plausibility questionnaire** consists of the same events as the LEI. Participants will rate “how plausible it is that the typical Pole who is about your age had certain childhood experiences by age 10” and will give their rating on a Likert scale from 1 (not at all plausible) to 8 (very plausible). As in the study by Sharman et al. [35], in order to avoid participants linking items from the LEI and plausibility scales to the personality questionnaires, the measures will be presented in a completely different layout.

**The procedure for inducing state memory distrust** consists of two stages and is administered *via* computer, similarly to Dudek & Polczyk [18]. Participants will be asked to recall eight life events that happened to them when they were less than 10 years old. They will briefly describe (1) what happened, (2) where it happened and (3) who took part in the event. This part of the procedure is based on the research of Winkielman et al. [97] and Merckelbach et al. [98], which has shown that remembering many events from childhood reduces the level of trust in one’s own memory abilities. Effort in the task is assumed to undermine one’s trust in one’s memory as participants attribute perceived difficulties during the task to the poor state of their own memory [97]. The second part of the procedure uses the premise that a key factor in MD is that giving false feedback makes people feel insecure [99]. A similar procedure has already been used by Szpitalak [100] that involves memorizing words from a list of 60 words in two minutes, then trying to remember as many words as possible. The computer screen will then display a false message which suggests that the participant performed poorly on this task compared to other participants.

The second method of inducing MD is rooted in the Barnum effect [101], which involves most people agreeing to descriptions of their personalities that are supposedly tailored specifically to them but are in fact vague and non-specific. The computer screen displays information about the “nature of memory processes”, e.g., “It is possible that you often experience the tip-of-the-tongue phenomenon when you know that you remember something but are unable to express it”. According to the Barnum effect, the manipulation will succeed if it causes participants to begin to doubt their memories.

In the control condition, participants will complete questionnaires unrelated to the purpose of the study. We decided to use a set of questionnaires measuring various kinds of susceptibility to influence, need for closure, and social desirability, which could be useful for exploratory analyses: the Gudjonsson Compliance Scale ([102]; Polish adaptation: [103]); The Inventory of Suggestibility ([104]; Polish adaptation: [105]); The Measure of Susceptibility to Social Influence ([106]; Polish adaptation: [36]); the Need for Closure Scale ([107]; Polish adaptation: [108]); and the Marlowe–Crowne Social Desirability Scale (MC, [109], Polish adaptation: [110]).

#### Manipulation check

Before and after the MD induction procedure, participants will complete a questionnaire containing four filler questions and one critical statement: “At the moment, I trust my ability to remember things”. Participants will indicate to what extent they agree with these statements (including the statement concerning memory distrust) using an analog visual scale, whose scores will be used in the analysis.

#### Personality questionnaires

***Squire Subjective Memory Questionnaire*** (SSMQ; [111]; Polish adaptation: [112]). This scale will be used to measure an aspect of memory distrust that is related to errors of omission, i.e., forgetting past experiences [17]. This questionnaire consists of 18 items rated on a nine-point scale, with answer options ranging from ‘disastrous’ (-4) to ‘perfect’ (4). The scale items include “My ability to search through my mind and recall names or memories I know are there is…” and “My ability now to remember what I read and what I watch on television is…” The internal consistency of the Polish version of SSMQ (Cronbach’s alpha) is .89, and the test-retest reliability is .87.

***Memory Distrust Scale*** (MDS; [17]). This scale will be used to capture memory distrust in relation to commission errors, i.e., remembering experiences that did not take place in the past. This tool contains 20 items, which are rated on a Likert scale ranging from “strongly disagree” (1) to “strongly agree” (7). The scale items include “I often look for physical evidence, such as photographs, to check whether things really happened the way I remember them” and “I believe some of my memories may have originated entirely from my imagination”. The total score ranges from 20 to 140, with higher scores suggesting greater levels of memory distrust. Internal consistency in validation studies was very high (Cronbach’s alpha ranging from .95 - .96), and test-retest reliability over a period of several weeks to 19 months was good. In the present study, a Polish translation of the MDS will be used.

***Rosenberg Self-Esteem Scale*** (SES; [113]; Polish adaptation: [114]. This measure of global self-esteem consists of 10 items (e.g., “At times I think I am no good at all”), to which participants respond on a four-point scale ranging from 1 (*I strongly agree*) to 4 (*I strongly disagree*). The Cronbach’s alpha of the Polish version of the SES is between .81 and .83. Its theoretical validity has been confirmed [114].

***Vividness of Visual Imagery Scale*** (VVIQ; [115]; Polish translation: Siuta, unpublished translation). This 16-item questionnaire measures imagery vividness. VVIQ involves having subjects imagine several suggested scenes. For each scenario, participants are asked to rate how vivid the image is using a 5-point scale, where “1” means “No image at all, I only ‘know’ I am thinking of the object”, “2” means “Dim and vague image”, “3” means “Moderately realistic and vivid”, “4” means “Realistic and reasonably vivid” and “5” means “Perfectly realistic, as vivid as real vision”. Example scenario: “Visualize a rising sun. Consider carefully the picture that comes to your mind’s eye”. An example of an item whose vividness is rated on the scale described above is “The sun rising above the horizon into a hazy sky”. Marks [115] obtained a test-retest reliability of .74 and a split-half reliability of .85.

***Verbalizer-Visualizer Questionnaire*** (VVQ; [116]; Polish translation: Siuta, unpublished translation). This self-report test comprises 30 questions and is designed to measure the extent to which people prefer to process information visually or verbally. Fifteen of the questions are diagnostic and refer to participants’ verbal and visual cognitive style. Each question is rated as “true” or “false”. Two dimensions are computed separately for the visual and verbal styles. An example of an item for visual ability: “My powers of imagination are better than average”). An example of an item for verbal ability: “I can easily think of synonyms for words”.

***Inventory of Childhood Memories and Imaginings*** (ICMI; [117]; Polish adaptation: [118]). This tool measures imagination immersion. The ICMI is a 52-item true-false questionnaire that is designed to assess the vividness of imagery, fantasy proneness, and strange experiences. The ICMI includes items such as “When I was younger, I enjoyed fairy tales” and “Now, I still live in a make-believe world some of the time”. Test-retest reliability and construct validity data are adequate, as documented in Lynn and Rhue [119].

***Tellegen Absorption Scale*** (TAS; [86]; Polish translation: Siuta, unpublished translation). This scale measures absorption, defined as the tendency to experience states of total attentional engagement. A version consisting of 37 self-descriptive statements with which the participant can agree or disagree will be used. An example item is “I like to watch cloud shapes change in the sky”.

***Dissociative Experiences Scale*** (DES; [120,121]. This scale measures the frequency of various interruptions and disturbances of consciousness, memory, and identity. In this study, we will use form C of the DES [122], in which participants rate how often they have each experience compared with other people. For example, participants are told “some people have the experience of finding themselves dressed in clothes that they don’t remember putting on” and are asked to “place a cross to show how much of the time this happens to you”. One end of the scale is labeled “much less than others”; the midpoint of the scale is “about the same as others”, and the other end is “much more than others”.

The total score of each of these questionnaires will be used in later analyses.

### Procedure

**Fig 2:**
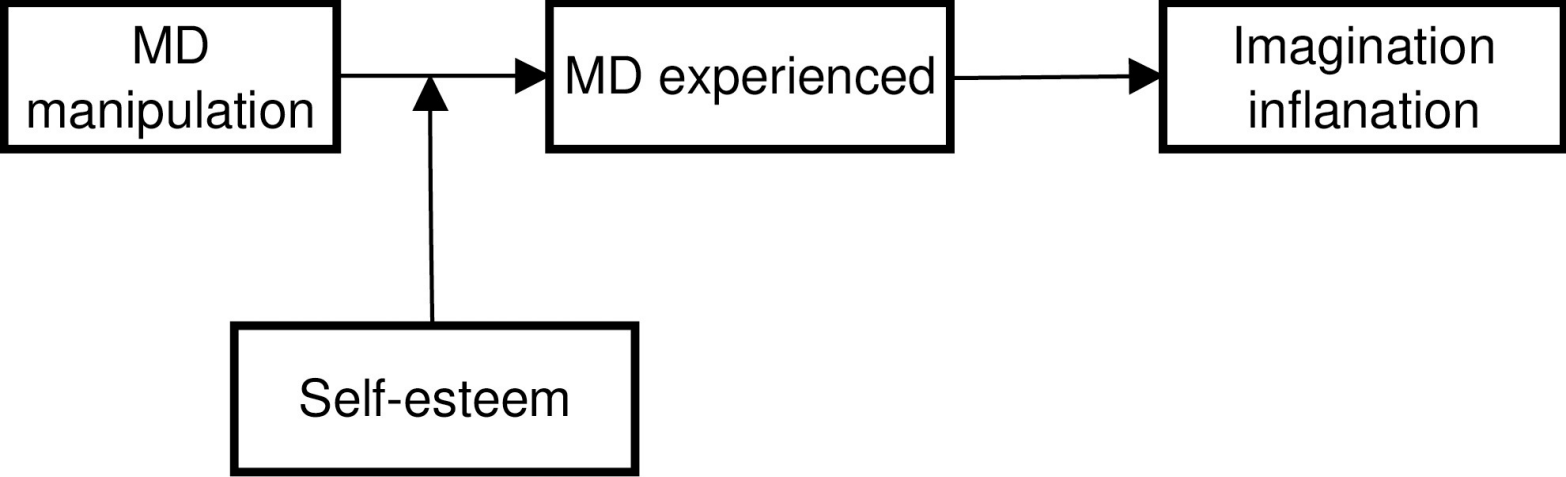
Flowchart of the experimental procedure.

The procedure will consist of three parts, as shown in Fig 2.

#### Part one

The first part will take place online. Participants will receive a link by e-mail to an online application, where they will complete the LEI 1 (pre-test), SSMQ and MDS questionnaires. It will take approximately 10 min to complete this stage, but no time limit will be imposed.

#### Part two

Approximately seven days after completing part one, participants will take part in the laboratory session, individually or in groups of 2 to 10 people. We will use a cover story similar to that of Sharman et al. (2005) [35], which states that participants will practice an imagination-based dream control technique. Each participant will sit at a computer workstation separated by an opaque wall from other participants. All instructions will be displayed on the computer screen. Participants will imagine three of the six target items from the LEI. The other three will serve as controls. Target events will be counterbalanced across subjects. Based on a guided imaginary instruction used in previous studies (e.g., [123, 124]), the instruction the participants receive will depend on the perspective from which they will imagine the events (see https://osf.io/dxf8g/?view_only=dfa8c06987824d6c9209cdfc11182f79 for detailed instructions). In the appropriate box on the screen, participants will describe what they have imagined about the event. Then they will fill in three personality questionnaires (VVIQ, IMCI and DES) in random order; this will take about 15 minutes, after which and they will be released. In order to maintain the plausibility of the cover story, participants will be given a bogus event to dream about (receiving a pet as a gift) and will be asked to imagine it before going to sleep at least twice before the next laboratory session.

#### Part three

Approximately seven days later, participants will complete the pre-test for MD manipulation and the remaining three personality questionnaires (SES, TAS and VVIQ in random order). Then, depending on the group, participants will either take part in the MD induction procedure or they will perform the tasks provided for the control group (in which there is no MD induction). After that, the post-test for MD manipulation will be applied. Subsequently, half of the participants will complete the post-test LEI (LEI 2) and then the plausibility questionnaire; the other half will complete the plausibility questionnaire and then LEI 2. These tools will be separated by a filler task. Finally, participants will answer questions concerning the purpose of the study and whether they have previously participated in research on memory and the psychology of witness testimony. At the end, participants will be debriefed.

### Proposed analysis

#### Manipulation check

A two-way ANOVA with one between-subjects factor (inducing memory distrust *vs*. the control group) × one within-subjects factor (mean results on VAS regarding trusting the memory before and after the manipulation). We will consider the manipulation effective when the decrease in memory trust will be statistically significantly greater in the experimental group than in the control one. Technically, this will be evidenced by a statistically significant interaction between the repeated measures factor and the between subjects factor, and the effects will be in the expected direction. Moreover, we will expect the magnitude of the change in mean memory distrust connected with the manipulation to be at least as great as half of the pooled mean standard deviation of the means. This would mean that the change is at least as much as one sten on the sten score.

#### Inflation score

We will use procedures based on those suggested by Garry et al. [125]. First, we will calculate the change score for each target event (imagined and not imagined) by subtracting confidence ratings at LEI 1 from confidence ratings at LEI 2. Then, we will subtract the change score for not-imagined events from the change score for imagined events. The final score is the mean change of imagined events (n = 3) minus the mean change of the not imagined (n = 3) events. A positive inflation score will indicate imagination inflation, while a negative score will indicate a decrease in confidence ratings.

#### Hypothesis 1

In order to verify Hypothesis 1a, the Bayesian approach will be applied, which allows the probability of the nonexistence of an effect to be determined. The Bayes factor for the Pearson *r* correlation will be computed and interpreted according to the guidelines proposed by van Doorn et al. [126]: a Bayes factor of less than 0.1 indicates strong evidence for the null hypothesis; a factor between 0.1 and 0.3 indicates moderate evidence for it; between 0.3 and 3.0 is inconclusive, and higher values support the alternative hypothesis. As for the prior in the Bayesian analysis, we are aware of no research confirming the relationship between MD and II. There is just one research at all concerning this [30] in which the impact of MD on II proved nonsignificant. Given this, the default prior was used, as recommended by van Doorn et al. [126]. Also, that robustness regions for Bayes factors will be provided.

JASP software [127] will be used for the calculations. In order to verify Hypothesis 1b, we will compute the Pearson correlation coefficient (*r*) between the imagination inflation score and the result of the MDS questionnaire. In order to verify Hypothesis 1b we will compute the Pearson correlation coefficient (*r*) between imagination inflation score and the result of the MDS questionnaire.

#### Hypothesis 2

A mixed 2 (memory distrust) × 2 (imagination) × 2 (source cue) × 2 (familiarity cue) repeated-measures ANOVA will be performed. Our hypothesis refers to comparisons of specific combinations of factor levels, so we will use planned comparisons [128–130]. We will report the results of the ANOVA for the main and interaction effects. When comparing the particular groups, we will base our comparison on simple effects.

We will check whether:

• II is greater in the group that is subjected to MD induction and receives the source cue but not the familiarity cue than in the group that is not subjected to MD induction and receives the same cue;

• II is greater in the group that is subjected to MD induction and receives the familiarity cue but not the source cue than in the group that is not be subjected to MD induction and receives the same cue;

• II is greater in the group that is subjected to MD induction and receives the familiarity cue and the source cue than in the group that is not subjected to MD induction and receives both these cues;

• II is greater in the group that is subjected to MD induction and receives the familiarity cue and the source cue than in the group that is subjected to MD induction and receives neither of these cues.

In general, it is assumed that providing two cues should increase the chances of participants noticing discrepancies and will sensitize them more than providing a single cue.

#### Hypothesis 3

A moderation analysis will be performed with a dichotomous predictor (induction *vs*. no induction of MD), a continuous moderator (a given indicator of disengaging from reality), and II as the dependent variable. Technically, such analyses consist in a multiple regression analysis with the predictor, moderator, and the product of the predictor and the moderator as independent variables. The significant effect of the product of the predictor and the moderator while controlling for both is considered to be an indication of a moderation. PROCESS 4.1 software will be used to perform the analyses [131]. Bootstrap-generated confidence intervals for the moderation effects will be used to determine statistical significance; if they do not include zero, the moderation will be considered significant. Separate analyses will be performed for each of the indicators of disengaging from reality.

#### Hypothesis 4

To analyze Hypothesis 4, moderated mediation will be analyzed, with self-esteem moderating the impact of the experimental manipulation that induces distrust on experienced distrust; the latter is a mediator of the impact of the manipulation of distrust on the imagination inflation effect. Technically, model **7** from the templates available in PROCESS 4.1 software will be used.

#### Data exclusion

Data will be excluded if any of the following criteria are met:

***LEI 1 and LEI 2 questionnaires*** (criteria based on previous research [55, 132])

1. The participant consistently rates all items as either 1 or 8 on either LEI 1 or LEI 2.
2. The participant provides a rating other than 1 on either LEI 1 or LEI 2 for any of the three unrealistic LEI events (i.e., “Won a million zlotys”, “Shook hands with the President”, “Played for Wisła Kraków”).

*Post-experimental interview*.

1. The participant correctly guesses the purpose of the study or the research hypothesis. The researcher will make a decision based on the answer to the question “What is the purpose of the research in your opinion (what does it investigate)?” It is planned to use three competent judges to determine whether what the subject said about the hypothesis is consistent with the hypothesis. Kendall’s W coefficient will be used to determine concordance among the judges.
2. The participant has already taken part in a study regarding memory distortion. The researcher will make a decision based on the description of the study provided by the participant. The exact question in the interview will be “Have you ever participated in a similar study?” (Choose either “yes” or “no”; if the participant selects “yes”, an additional window will appear requesting a description of what the study entailed.).
3. The participant reports technical problems (when asked to comment on the study procedure or during the procedure itself).
4. The participant answers “No” to the seriousness check question: “Did you give honest and truthful answers to the questions? Did you approach the study survey seriously? Your answer to this question will not affect your reward for participating in the survey.

*Attention check*. The participant fails at least one of the three attention checks. These will be items hidden among personality questionnaire questions, such as “Please select ‘very often’ (this is to check your attention) with the response options from *(1) never* to *(4) very often*.

*Adherence to the cue of perspective for imagining events*. The researcher will analyze the imagery descriptions generated by the participants. If the perspective does not match the one to which the person was assigned in at least one (out of three) descriptions provided by the participant, the data from this participant will be excluded.

These data will be replaced by data from other participants to achieve the required sample size.

#### Missing data

The use of computerized questionnaires ensures there will be no missing data because a participant cannot proceed to the next part of the study if they do not provide an answer to a given question. If a participant misses the in-lab phase of the study, another person will be recruited to take their place.

#### Timeline for completion of the study

The experiment will be conducted in 2024. If the Stage 1 reviews are accepted, the resubmission should be done no later than 2025.

## Supporting information

S1 File Power analysis. Screenshots from G*Power.
(DOCX)
