# Supplementary material for: Memory distrust and imagination inflation: A registered report
Source: PLoS One. 2024 Feb 7;19(2):e0297774. doi: 10.1371/journal.pone.0297774 (PMC10849220; doi:10.1371/journal.pone.0297774)

**ANOVA Interaction large effect**


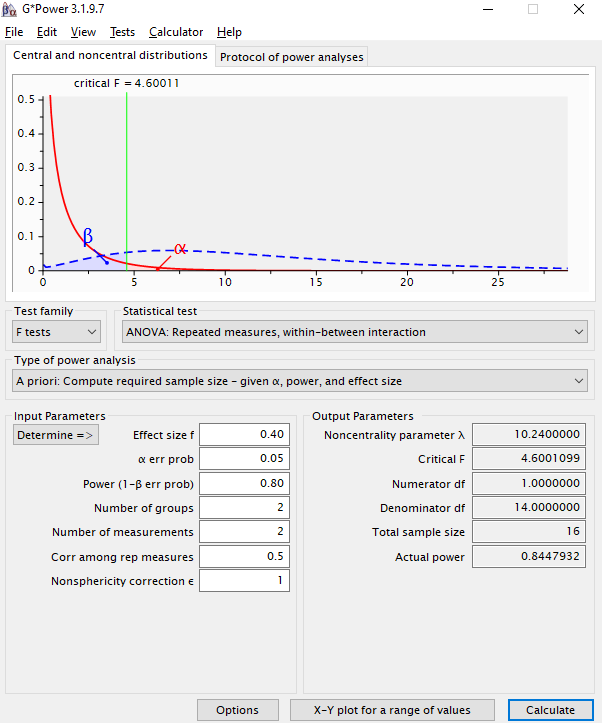


**ANOVA Interaction medium effect**


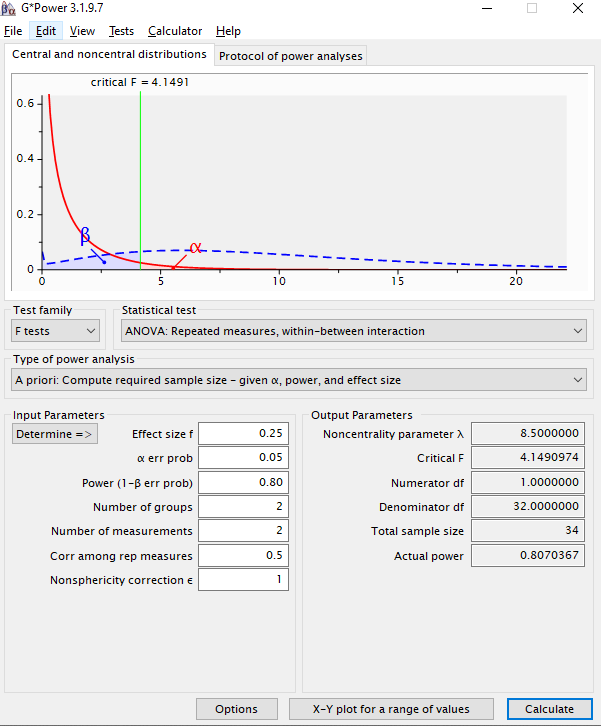


**ANOVA Interaction small effect**


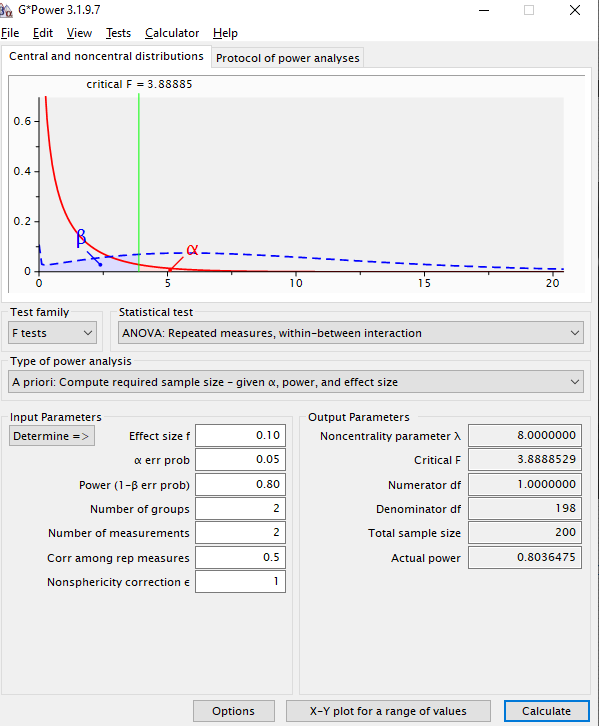


**Moderation large effect**


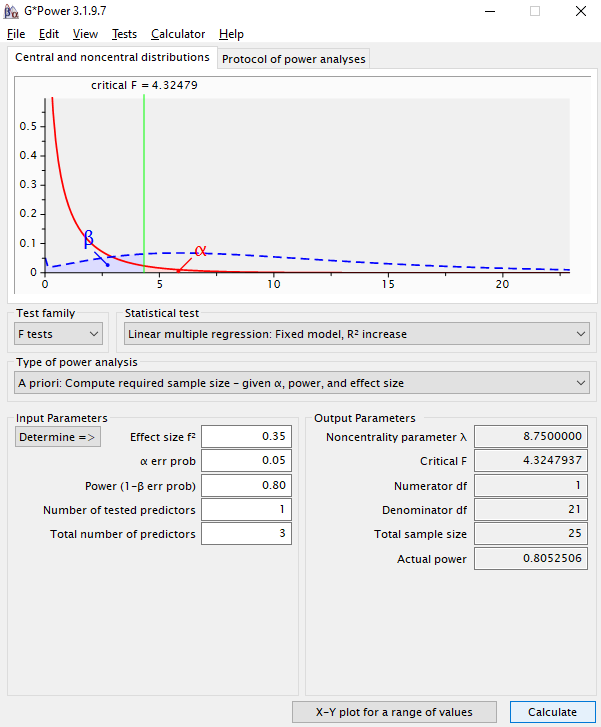


**Moderation medium effect**


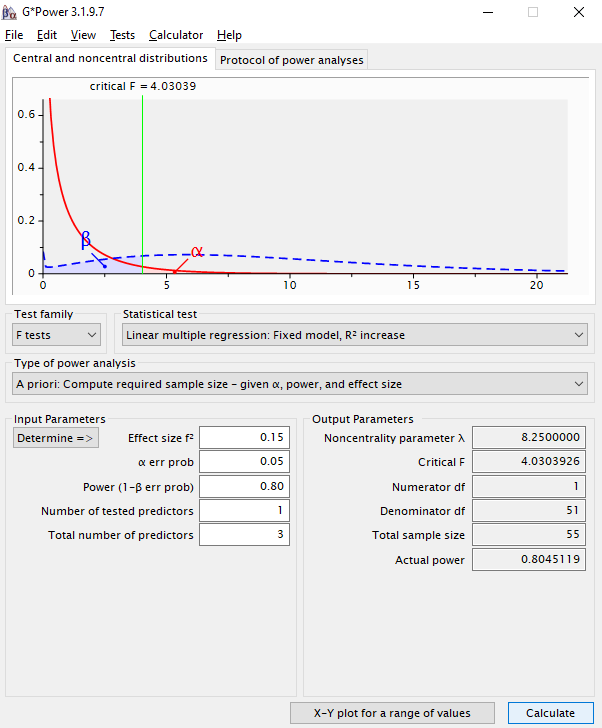


**Moderation small effect**


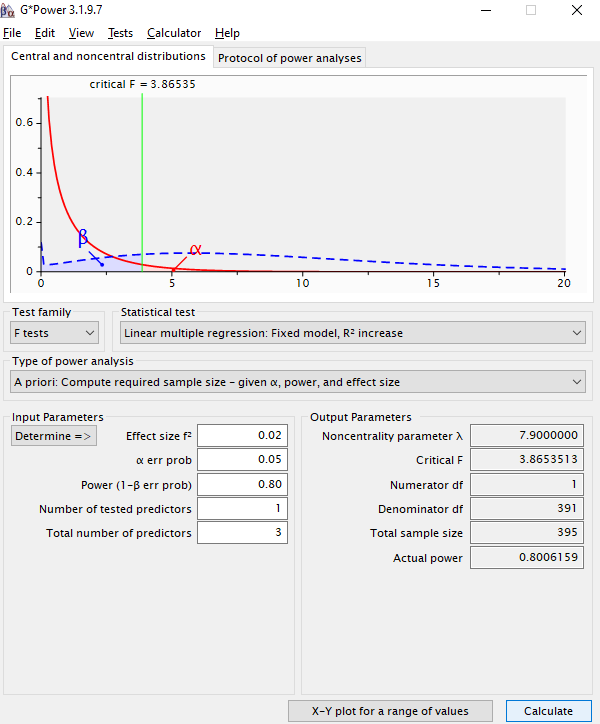


**Repeated ANOVA large effect**


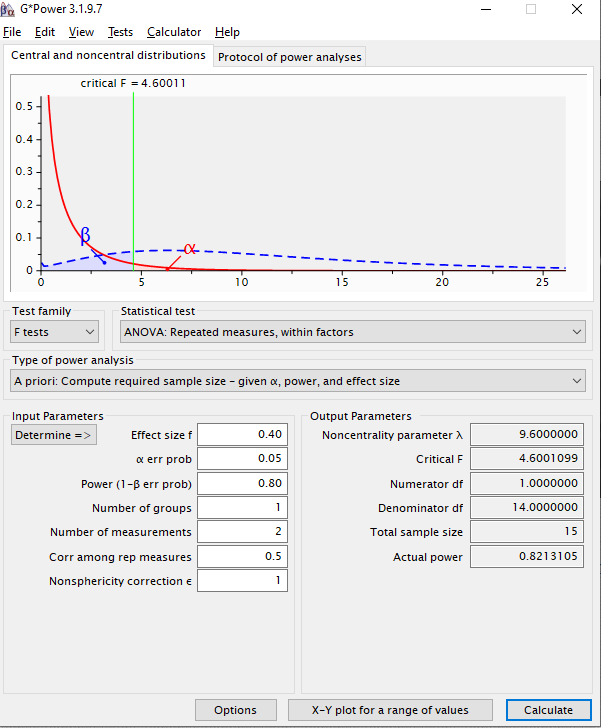


**Repeated ANOVA medium effect**


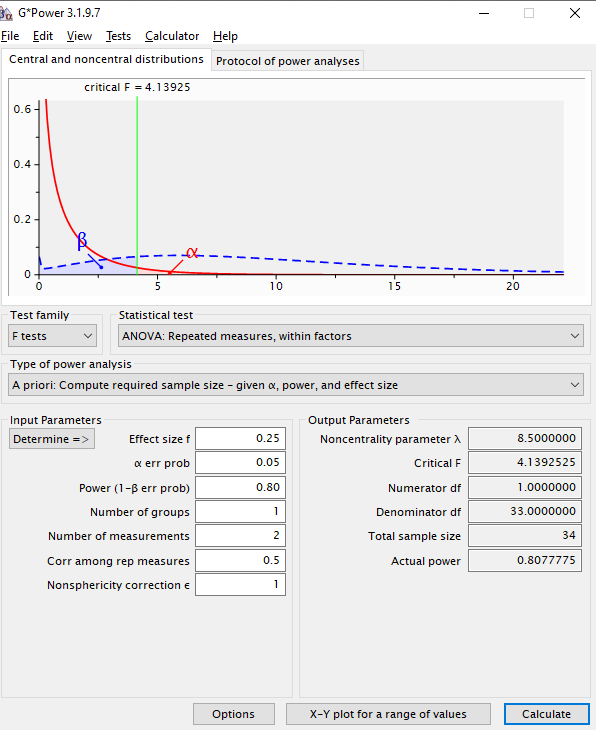


**Repeated ANOVA small effect**


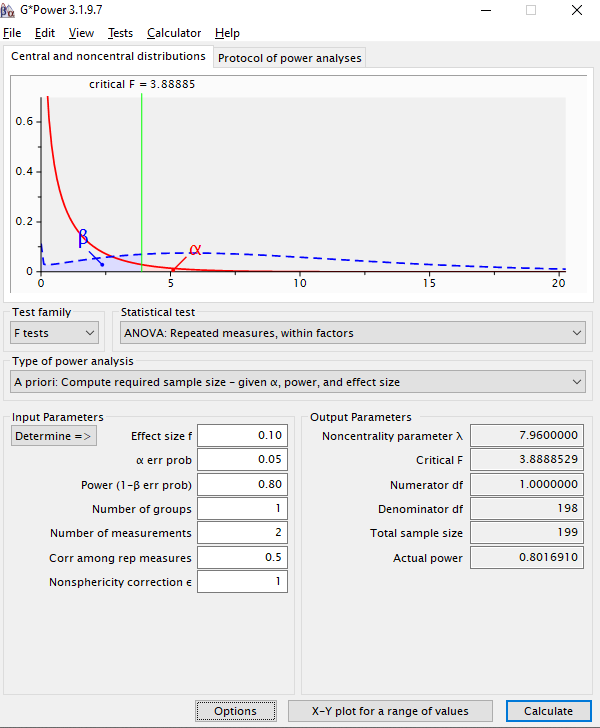

Supplement: S1 File — Screenshots from G*Power. (DOCX) [file pone.0297774.s001.docx]
